# Alzheimer's early detection in post-acute COVID-19 syndrome: a systematic review and expert consensus on preclinical assessments

**DOI:** 10.3389/fnagi.2023.1206123

**Published:** 2023-06-21

**Authors:** Clair Vandersteen, Alexandra Plonka, Valeria Manera, Kim Sawchuk, Constance Lafontaine, Kevin Galery, Olivier Rouaud, Nouha Bengaied, Cyrille Launay, Olivier Guérin, Philippe Robert, Gilles Allali, Olivier Beauchet, Auriane Gros

**Affiliations:** ^1^Institut Universitaire de la Face et du Cou, ENT Department, Centre Hospitalier Universitaire, Nice, France; ^2^Laboratoire CoBTeK, Université Côte d'Azur, Nice, France; ^3^Centre Hospitalier Universitaire de Nice, Service Clinique Gériatrique du Cerveau et du Mouvement, Nice, France; ^4^Département d'Orthophonie, UFR Médecine, Université Côte d'Azur, Nice, France; ^5^Institut NeuroMod, Université Côte d'Azur, Sophia Antipolis, France; ^6^ACTLab, engAGE: Centre for Research on Aging, Concordia University Montreal, Montreal, QC, Canada; ^7^Research Centre of the Geriatric University Institute of Montreal, Montreal, QC, Canada; ^8^Leenaards Memory Center, Lausanne University Hospital and University of Lausanne, Lausanne, Switzerland; ^9^Federation of Quebec Alzheimer Societies, Montreal, QC, Canada; ^10^Mc Gill University Jewish General Hospital, Montreal, QC, Canada; ^11^Université Côte d'Azur, CNRS UMR 7284/INSERM U108, Institute for Research on Cancer and Aging Nice, UFR de Médecine, Nice, France; ^12^Departments of Medicine and Geriatric, University of Montreal, Montreal, QC, Canada

**Keywords:** Alzheimer's disease, post-acute COVID-19 syndrome, biomarkers, early diagnosis, olfactory disorders

## Abstract

**Introduction:**

The risk of developing Alzheimer's disease (AD) in older adults increasingly is being discussed in the literature on Post-Acute COVID-19 Syndrome (PACS). Remote digital Assessments for Preclinical AD (RAPAs) are becoming more important in screening for early AD, and should always be available for PACS patients, especially for patients at risk of AD. This systematic review examines the potential for using RAPA to identify impairments in PACS patients, scrutinizes the supporting evidence, and describes the recommendations of experts regarding their use.

**Methods:**

We conducted a thorough search using the PubMed and Embase databases. Systematic reviews (with or without meta-analysis), narrative reviews, and observational studies that assessed patients with PACS on specific RAPAs were included. The RAPAs that were identified looked for impairments in olfactory, eye-tracking, graphical, speech and language, central auditory, or spatial navigation abilities. The recommendations' final grades were determined by evaluating the strength of the evidence and by having a consensus discussion about the results of the Delphi rounds among an international Delphi consensus panel called IMPACT, sponsored by the French National Research Agency. The consensus panel included 11 international experts from France, Switzerland, and Canada.

**Results:**

Based on the available evidence, olfaction is the most long-lasting impairment found in PACS patients. However, while olfaction is the most prevalent impairment, expert consensus statements recommend that AD olfactory screening should not be used on patients with a history of PACS at this point in time. Experts recommend that olfactory screenings can only be recommended once those under study have reported full recovery. This is particularly important for the deployment of the olfactory identification subdimension. The expert assessment that more long-term studies are needed after a period of full recovery, suggests that this consensus statement requires an update in a few years.

**Conclusion:**

Based on available evidence, olfaction could be long-lasting in PACS patients. However, according to expert consensus statements, AD olfactory screening is not recommended for patients with a history of PACS until complete recovery has been confirmed in the literature, particularly for the identification sub-dimension. This consensus statement may require an update in a few years.

## 1. Introduction

Since the beginning of the COVID-19 pandemic, many patients remain impaired in their daily life, long after the infection. In a study based upon an international cohort (Davis et al., 2021), cognitive, sensory-motor, memory, and speech or language symptoms persisted in an average of 30% (Ceban et al., 2022; d'Ettorre et al., 2022; Han et al., 2022; Nehme et al., 2022) of patients up to 7 to 12 months after SARS-CoV-2 infection (COVID-19). These symptoms are grouped under the term of Post-Acute COVID-19 Synonym (PACS) as defined by an OMS Delphi consensus^1^. Morphological MRI changes in brain structure have also been observed for approximately 141 days after the infection (Douaud et al., 2022) including primarily in global brain size and, secondarily in a decrease of the olfactory cortex thickness. Major changes in tissue damage markers in brain areas functionally connected to the primary olfactory cortex were also observed (Douaud et al., 2022), which could explain why 29.8% of PACS patients complain of persistent dysosmia, or a change in the sense of smell, more than 24 months after COVID-19 (Lechien et al., 2023). The point is that much of the recent literature focuses on the emerging risk of neurodegenerative disease and more precisely on AD (Luukkainen et al., 2018; Heneka et al., 2020; Rebholz et al., 2020; Verkhratsky et al., 2020; Erausquin et al., 2021; Mahalaxmi et al., 2021; Beauchet and Allali, 2022; Chen et al., 2022) after contracting COVID-19.

Worldwide, Alzheimer's disease (AD) is the main neurodegenerative disease leading to dementia, and it is responsible for an increase in morbidity (Scheltens et al., 2021) affecting more than 50 million people, two-thirds living in low- and middle-income countries (Scheltens et al., 2021). The prevalence of AD is estimated to triple in 2050 (Scheltens et al., 2021). Preclinical and prodromal AD respectively lasts on average for 10 and 4 years (Vermunt et al., 2019) before becoming dementia. The median survival rate for dementia is approximately about 3–6 years (Mayeda et al., 2017; Rhodius-Meester et al., 2019) after diagnosis. AD early diagnosis, followed by non-pharmacological interventions and pharmacological treatment (Scheltens et al., 2021), could potentially stall the rapid cognitive decline associated with dementia. However, early diagnosis remains a real challenge for clinicians as preclinical AD screening tests are still debated.

Current conventional and preclinical AD screening markers, such as neuropsychological assessments, brain morphological (MRI) or metabolic (PET), or a lumbar puncture for example (Drago et al., 2011; Scheltens et al., 2021), are not equally available worldwide. They are expensive, time-consuming, and depend on the availability of both technological platforms and human assistance. Remote digital Assessments for Preclinical Alzheimer's disease (RAPAs) could be an alternate solution that is relatively easy to implement and which might reduce delays in preclinical AD diagnoses. During the COVID-19 pandemic, remote assessments became increasingly common in daily medical practice with telemedicine enabling patients to benefit from continuous remote monitoring through a variety of digital technologies, such as video conferencing tools or symptom tracking applications (Beauchet et al., 2020). Remote assessments, both to provide cognitive assessment and plan treatment interventions, allow patients to have easier access to specialists and highly skilled healthcare professionals—even if those patients are located in remote regions—in a feasible, effective, and acceptable way (Poon et al., 2005; Sekhon et al., 2021). Furthermore, telemedicine is part of an approach to technology use that is intergenerational and has included a growing number of older adults (Fraser et al., 2020) which increased during the pandemic. Telemedicine has been found to help patients to avoid unnecessary travel and limit hospitalizations, which may be desired by some patients and reduce the costs of managing diseases. However, telemedicine is also a challenging process based on a number of different factors including access and ownership of the appropriate digital tools, the ability to use these tools, the physical affordances of the devices and the mobility of the patient, as well as interactional barriers in communicating digitally with someone in a health crisis (Dassieu et al., 2022).

A group of AD remote and digital evaluation platform experts from France, Switzerland, and Canada (IMPACT project) under the leadership of the French National Research Agency, were invited to develop evidence-based recommendations and expert consensus on items related to AD early diagnosis in the post-COVID-19 era. The IMPACT project aims to: 1- review RAPAs potentially impaired in PACS patients which could become unusable in AD early screening in case PACS last a long or a lifetime; 2- describe evidence-based recommendations according to the review; 3-inform people and policymakers of the recommendations. The first and the second items are the primary and secondary objectives of this work.

## 2. Methodology

### 2.1. Search strategy

#### 2.1.1. Selection of the remote digital assessments for preclinical Alzheimer's disease

**Figure 1:**
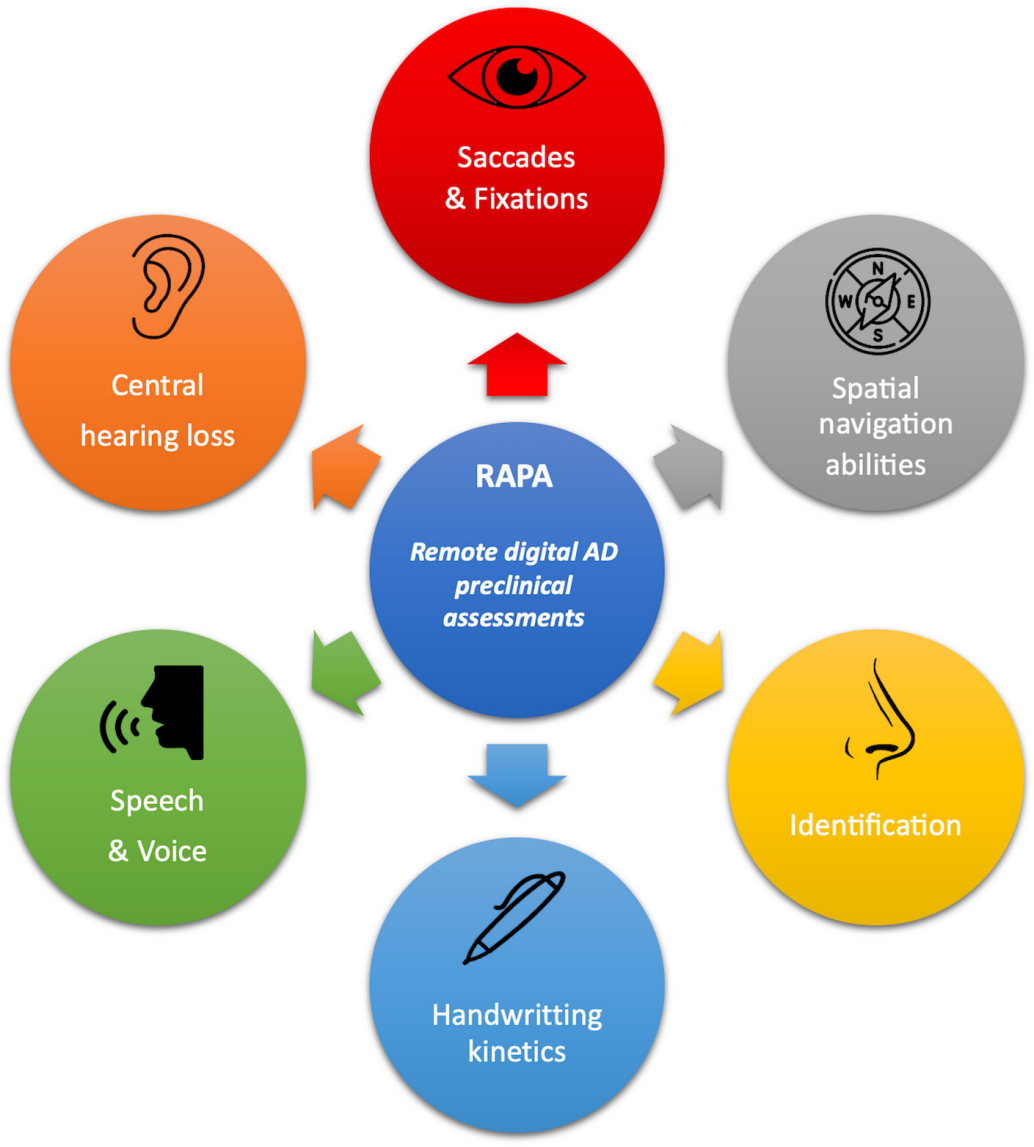
Overview of all RAPAs and their main clinical target assessments. Every detail of these RAPAs and the literature review process we used to summarize them is provided in Supplementary Data Sheet 2.

An initial research stage allowed authors to identify Remote digital Assessments for Preclinical AD (RAPAs) in PubMed and Embase Databases which were easily usable, non-expansive, quick, and widely available: vocal, graphical, eye tracking, central auditive impairments, olfactory disorders, and spatial navigation abilities markers were all assessed. A complete process is reported in Supplementary Data Sheet 2 and all selected RAPAs are illustrated in Figure 1.

#### 2.1.2. Data sources

A search request command on PubMed, Cochrane database, and Embase was entered on 31/11/2022. This search included “*keywords*” through VOCAL “speech” OR “language” OR “language tests” OR “voice”; GRAPHICAL “Psychomotor Performance” OR “writing” OR “handwriting” OR “psychomotor performance” OR “mouse movements” OR “patterns” OR “drawing” OR “keystroke”; EYE TRACKING “eye movement” OR “eye-tracking technology” OR “saccades” OR “ocular motility”; CENTRAL AUDITIVE IMPAIRMENTS “Auditory system dysfunction” OR “central auditory function” OR “central auditory deficit”; OLFACTORY DISORDERS “Olfaction Disorders” OR “anosmia” OR “hyposmia” OR “dysosmia” OR “olfactory loss” OR “parosmia”; SPATIALIZATION “Virtual reality” OR “spatial navigation”. PACS included “long covid”, “post covid”, “post-covid, “post-covid-19”, “long-covid-19”, “long-covid” or “post-acute covid-19 syndrome”. The search request strategy is provided in Supplementary Data Sheet 1.

### 2.2. Inclusion criteria

#### 2.2.1. Types of studies

Systematic reviews with or without meta-analyses and observational studies (only those in peer-reviewed journals) were included. We excluded retrospective studies, meeting abstracts, conference presentations, book reviews, news items, and corrections. Every study in English, relative to humans since 2020, was included if they were a clinical trial, a meta-analysis, a randomized controlled trial, a review, or a systematic review. Studies in languages other than English or French, older than 2020, or without abstract were not included as the COVID-19 pandemic began that year. The electronic database search was supplemented by screening the reference lists of the included studies and relevant reviews.

#### 2.2.2. Types of participants

Only adult (≥18 years old) patients with post-acute COVID-19 syndrome (PACS) were included, but this term is not always called PACS but sometimes “long-COVID-19” or “post-COVID-19”. These terms were included in the search strategy protocol. Exclusion criteria were patients previously impaired with neurologic, neurodegenerative, or neuromuscular diseases; speech, voice, or language impairments; psychomotricity, writing- or handwriting-related diseases; abnormal eye-movement related diseases; anterior reported hearing loss; anteriorly reported olfaction disorders or spatial navigation incapacities. All types of intervention were included.

#### 2.2.3. Types of outcomes

We determined that outcome measures must include one or more of the RAPA previously identified among vocal, graphical, eye tracking, central auditive impairments, olfactory disorders, or spatial navigation impairment.

#### 2.2.4. Study selection and evaluation

For the first step, two reviewers (CV, AP) assessed the title/abstract of each result following inclusion and exclusion criteria. In case of conflict, a second review was scheduled with both reviewers (CV, AP) and a third (AG) until a consensus was reached. Individual clinical research studies were evaluated in accordance with the French HAS criteria.

#### 2.2.5. Quality assessment

The quality of the studies reported was assessed based on a systematic review of methodological quality assessment tools (Zeng et al., 2015). Systematic reviews were assessed using the AMSTAR 2 tool (Assessment of Multiple SysTemAtic Reviews) (Shea et al., 2007, 2017), cohort and observational studies using the Observation Study Quality Evaluation tool (OSQE) (Drukker et al., 2021). Concerning AMSTAR 2, 16 items were evaluated and of these 7 were critical (N°2, 4, 7, 9, 11, 13, 15). A review was assessed as high quality if none or one non-critical weakness was noticed (the systematic review provides an accurate and comprehensive summary of the results of the available studies that address the question of interest). A review was assessed as moderate quality when more than one non-critical weakness was noticed (the systematic review has more than one weakness but no critical flaws. It may provide an accurate summary of the results of the available studies that were included in the review). Reviews were assessed as low quality when one critical flaw with or without non-critical weaknesses were noticed (the review has a critical flaw and may not provide an accurate and comprehensive summary of the available studies that address the question of interest). Finally, reviews were assessed as critically low-quality when more than one critical flaw with or without non-critical weaknesses (the review has more than one critical flaw and should not be relied on to provide an accurate and comprehensive summary of the available studies) or multiple non-critical weaknesses were noticed (may diminish confidence in the review and it may be appropriate to move the overall appraisal down from moderate to low confidence). For the OSQE evaluation tool, 15 items were evaluated with different evaluation weights explaining why authors (Drukker et al., 2021) did not provide any cut-off score to discriminate good from poor quality studies. Different forms were used, provided by authors in their original work (Drukker et al., 2021), given depending on the observational study type. No meta-analysis was done, so the risk of bias associated with the included studies was not assessed. Ethical clearance from the institutional ethical committee was not required as all the data extracted was from already published studies and no patients or the public were directly interviewed or involved in the present research.

### 2.3. Consensus process

The EU Joint Program—Neurodegenerative Disease Research (JPND) initiative initiated a call for expert working groups on 1 November 2021, to investigate the impact of COVID-19 on research related to neurodegenerative diseases. In response to the program's call, various national funding organizations were asked to participate based on the country's response. The Funding organization from France, for example, was the French National Research Agency. Our working groups answered this call, which focused on the COVID-19 pandemic and its impact on Alzheimer's care. This included setting up an expert board based on past collaborations in this field of expertise. Talking about digital and clinical distance evaluation platforms required bringing together other specialists in the field of digitalization and digital support explaining working with physicians (CV, OR, CyLa, OG, PR, GA, OB) neuroscientists (AP, VM, KG, NB, OG, PR, GA, OB, AG), speech therapists (AP, AG), communication and age studies experts (CoLa and KS) and a social media research director (KS). VM and AG developed the research topics using the population, intervention, comparator, and outcome (PICO) framework and created the initial recommendation statements. In the first round, a group of 12 experts from the IMPACT project reviewed and provided feedback on the questionnaire using a 5-point scale (ranging from “strongly agree” to “strongly disagree”) (Bossard et al., 2018). Responses with a score of 1–2 were considered as indicating agreement. During the second round, the recommendation statements that did not achieve agreement were discussed further. If a consensus agreement of 75% was not reached after discussion, a third round of rating was conducted (Sanz-Paris et al., 2017). Finally, the grades of recommendation were assigned based on the strength of evidence and a consensus discussion of the results from the Delphi rounds.

## 3. Results

### 3.1. Literature search results

**Figure 2:**
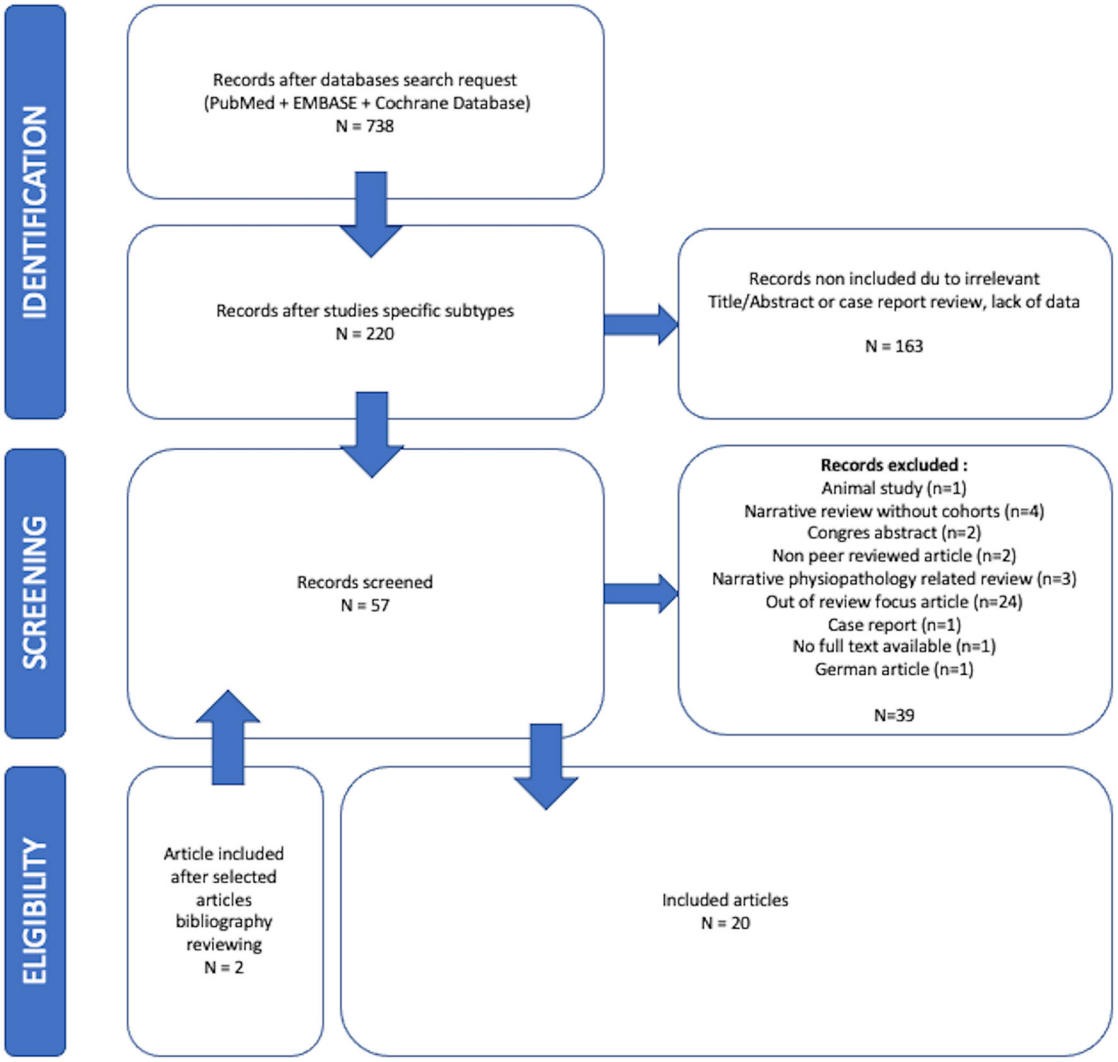
Overview of the screening process. PRISMA flow diagram of the studies selection process.

The initial research team (CV, AP, AG) reviewed 738 articles. Twenty studies met the inclusion criteria after evaluation of titles, abstracts, and full contents of the relevant studies of which four were systematic reviews, seven systematic reviews with meta-analysis, two narrative reviews, six observational cohorts, and one case-control study. The entire selection process is reported in a flowchart (Figure 2). The reviews and observational studies' level of quality are reported in Tables A, B, and C in the Supplementary Data Sheet 3. Based on AMSTAR 2 scores 69.2% (*n* = 9), 23.1% (*n* = 3), and 1.8% (*n* = 1) reviews had respectively critically low, low, and high quality. Based on OSQE scores, 14.3% (*n* = 1), 28.6% (*n* = 2), 42.9% (*n* = 3), and 28.6% (*n* = 2) of observational studies were, respectively, scored with 14, 13, 12, and 8 stars out of 15.

#### 3.1.1. Demographical data

Demographics are reported in Table 1. When it was reported (90%; *n* = 18) population size ranged from 34 (Vandersteen et al., 2021) to 178 496 (Parker et al., 2021) people with an average of 24 031 ± 54,301 patients in reviews papers and 638 ± 1,379 in cohort observational studies papers. Patients' ethnic groups were reported to be from all over the world apart from five (38.5%) reviews (Deer et al., 2021; Bertuccelli et al., 2022; De Luca et al., 2022a; Jamoulle et al., 2022; Premraj et al., 2022) and five (71.4%) observational studies (Vandersteen et al., 2021; Girón Pérez et al., 2022; Mendes Paranhos et al., 2022; Michelutti et al., 2022; Ser et al., 2022) where patients where reported to come from only one country depending on teams origins. When it was clearly reported in 50% of studies (*n* = 10), the average patient ages were 40- and 50-years-old. Only 12 studies clearly reported gender impairment differences with women preferentially impaired in 6/12 studies. The definition of long-COVID-19 has changed a lot in the past 2 years and so heterogeneous assessment time from COVID-19 onset was reported in Table 1.

**Table 1:** Main demographic data included in the selected articles.

**Authors**	**Time from COVID-19 onset**	***N* (patients)**	***N* review (studies)**	**Type**	**Age [years ±SD; (min-max)] ~in average**	**Women *N* (%)**
(Ahmad et al., 2021)	2 weeks to 6 months	14,056	20	SR	(18–60)	-
(Parker et al., 2021)	2 weeks to 6 months	178,496	272	SR	(17–93)	-
(Ser et al., 2022)	4 weeks to 3 months	106	-	CCS	39.4 ± 12.5	47 (44.3)
(Deer et al., 2021)	17 days to 4,7 months	NP	59	SR/MA	(12–73)	-
(Bertuccelli et al., 2022)	3–6 months	1,940	25	SR	42.57 ± 7.23 to 79 ± 8; ~60	873 (45)
(Dirican and Bal, 2022)	23 days to 12 months	7,546	20	SR/MA	53.4 ± 8.2; (34–68.8)	1,671 (46.8)
(Jamoulle et al., 2022)	3–18 months	55	-	CS	42,9 ± 15,6	40 (72.7)
(De Luca et al., 2022a)	1 month to 10,6 months	5,582	16	SR	-	-
(Davis et al., 2021)	0–7 months	3,762	-	CS	(30–60)	2,969 (78.9)
(Silva Andrade et al., 2021)	NP	NP	62	NR	-	-
(Premraj et al., 2022)	3–6 months	10,530	18	SR/MA	52 ± 10	6,213 (59)
(Pinzon et al., 2022)	Until 6 months	9,944	36	SR/MA	(17–81)	-
(Malik et al., 2022)	30–180 days	4,828	12	SR/MA	~58.75	2,481 (45,5)
(Girón Pérez et al., 2022)	More than 3 months	76	-	CS	~45 (20–70)	36 (47.4)
(Xydakis et al., 2021)	47 days to 6 months	3,691	-	NR	-	-
(Fernández-de-Las-Peñas et al., 2021)	0–3 months	24,225	33	SR/MA	47.8 ± 16.6	52.26
(Tan et al., 2022)	0–6 months	3,699	18	SR/MA	(30–55.8)	-
(Michelutti et al., 2022)	More than 3months	213	-	CS	53 ± 14	151 (73)
(Mendes Paranhos et al., 2022)	221–264 days	219	-	CS	(18–60)	164 (74.9)
(Vandersteen et al., 2021)	5 ± 2,8 months	34	-	CS	41.6 ± 12.9	16 (47)

SD, Standard deviation; SR, systematic review; CCS, case control study; SM/MA, systematic review and meta-analysis; NR, narrative review; CS, cohort study.

#### 3.1.2. Impairments observed in the RAPA patients

The summary results of RAPAs review were reported in Table 2. We independently analyzed RAPA impairments in 20 studies and reported direct or indirect impairment for each RAPA (summarized in Table 3) as RAPA could have been directly impaired (for example hand shaking in handwriting assessments) or indirectly impaired (like visual hallucinations in eye-tracking assessments). The most often-reported RAPA impairment was the olfactory function occurring in PACS patients in all but two studies. The second most frequently impaired biomarkers were graphical and eye-tracking ones. The third was central hearing and finally vocal and spatial navigation abilities were reported very rarely.

**Table 2:** Remote digital Alzheimer's disease preclinical assessments (RAPA) impairments related to every study included in the review.

	**RAPA**					
**Authors**	**Vocal**	**Graphical**	**Eye tracking**	**Central hearing**	**Olfactory disorders**	**Spatial navigation**
(Ahmad et al., 2021)	X	X	X		X	
(Parker et al., 2021)				X	X	
(Ser et al., 2022)		X			X	
(Deer et al., 2021)	X	X	X	X	X	
(Bertuccelli et al., 2022)						X
(Dirican and Bal, 2022)					X	
(Jamoulle et al., 2022)		X				
(De Luca et al., 2022a)				X	X	
(Davis et al., 2021)	X	X	X	X	X	
(Silva Andrade et al., 2021)	X	X	X	X	X	
(Premraj et al., 2022)					X	
(Pinzon et al., 2022)		X	X	X	X	X
(Malik et al., 2022)					X	
(Girón Pérez et al., 2022)					X	
(Xydakis et al., 2021)					X	
(Fernández-de-Las-Peñas et al., 2021)			X		X	
(Tan et al., 2022)					X	
(Michelutti et al., 2022)			X		X	
(Mendes Paranhos et al., 2022)					X	
(Vandersteen et al., 2021)					X	
Total number of studies	4	7	7	6	18	2

**Table 3:** Recommendations summary related to Remote digital Alzheimer's disease preclinical assessments (RAPA) evaluated in the review.

**Assessed items**	**A**	**B**	**C**	**Level of evidence**	**Grade ofrecommendation**
	**AD early diagnosis RAPA interest**	**AD RAPA specificity loss in PACS patients**	**RAPA interest in early diagnosis of AD in PACS patients**		
Vocal markers	**4.3** **±0.7**	3.7 ± 0.9	3.9 ± 0.9	II	B
Graphical markers	**4.1** **±0.8**	3.9 ± 1	3.9 ± 0.6	II	B
Eye-tracking	2.7 ± 0.7	3.3 ± 0.7	3.3 ± 0.9	II	B
Central hearing	2.6 ± 0.5	3.1 ± 0.8	3 ± 0.5	II	B
Olfactory disorders	**4** **±0.9**	1.6 ± 1.4	1.4 ± 0.7	II	B
Spatial navigation abilities	3.7 ± 1	3.7 ± 1.3	3.7 ± 1.5	II	B

AD, Alzheimer's disease; RAPA, Remote digital Assessments for Preclinical AD; PACS, post-acute COVID-19 syndrome. Likert scale for items A and C are reported as 1 for not agreeing at all to 5 for completely agreeing. For item B (biomarker specificity loss in PACS patients), Likert scale was inverted. Results in bold are related to impacted likert results for Delphi methodology (≥4).

Summary of the expert' recommendations for the use of every RAPA in PACS are reported in Table 3.

### 3.2. Consensus recommendations

Many RAPAs were reported as impacted in PACS patients but olfaction was the most impaired. Graphical and eye-tracking assessments were fewer but still reported as impacted. Consensus recommendations were discussed based on these reports.

#### 3.2.1. Consensus recommendation: olfaction-related remote digital assessments for preclinical Alzheimer's disease

Olfaction was impacted in all but two studies (Bertuccelli et al., 2022; Jamoulle et al., 2022). Direct involvement included a persistent dysosmia in 11–57.6% of PACS patients (Ahmad et al., 2021; Davis et al., 2021; Parker et al., 2021; Silva Andrade et al., 2021; Girón Pérez et al., 2022; Michelutti et al., 2022; Tan et al., 2022) related to an anosmia (Ahmad et al., 2021; Fernández-de-Las-Peñas et al., 2021; Malik et al., 2022; Mendes Paranhos et al., 2022; Michelutti et al., 2022; Premraj et al., 2022; Tan et al., 2022), explicitly reported in 12.8% (Deer et al., 2021), 19.3–21.4% (Ahmad et al., 2021), 32.2% (Michelutti et al., 2022), 44% (Ser et al., 2022), or 55.9% (Vandersteen et al., 2021) of cases or hyposmia (Mendes Paranhos et al., 2022; Michelutti et al., 2022) explicitly reported in 14.7% (Vandersteen et al., 2021), 15.3% (Deer et al., 2021), or 33.1% (Michelutti et al., 2022)of cases. Assessment time from COVID-19 onset was extremely variable. Only two studies were over 10 to 12 months of follow-up (De Luca et al., 2022a; Dirican and Bal, 2022) but only De Luca et al. (De Luca et al., 2022a) report a 6-month recovery rate of 95.3% in a 16 study review on PACS persistent chemosensory dysfunction. One study (Vandersteen et al., 2021) reported olfaction subdimensions precisions related to a prevalent identification impairment significantly related to subjective olfactory recovery (VAS; *p* = 0.034) compared to threshold and discrimination scores. According to WHO clinical management of COVID-19,^2^ Dirican and Bal (2022) did not find any difference in the persistence of anosmia between severe and non-severe survivors of COVID-19 with a global pooled odds ratio of 1.22 (95%CI 0.69 to 2.16) in a meta-analysis of 20 relevant observational studies. Parosmia was explicitly reported in 23.2% (Davis et al., 2021) and frequently described as “smoke,” “burning,” “cigarette,” and an altered “meat” smell. Phantosmia was reported in 23.2% (Deer et al., 2021). Indirect involvement included dysgeusia (Ahmad et al., 2021; Fernández-de-Las-Peñas et al., 2021; Pinzon et al., 2022; Premraj et al., 2022), which was frequently reported associated with olfaction disorders in 19.3–38.5%of the studies (Davis et al., 2021; Deer et al., 2021; Silva Andrade et al., 2021). Davis et al. (2021) found no significant differences between loss of smell [35.9%, (34.4–37.5%)] vs. loss of taste [33.7%, (32.2–35.2%), *p* > 0.1] in an online questionnaire observational study on 3762 PACS patients 7 months after COVID-19 onset. More precisely, parageusia and phantageusia, similar to qualitative olfactory dysfunction, were reported respectively in 16.4 and 9% (Deer et al., 2021) of PACS patients up to ~5 months after COVID-19 onset.

Considering above discussion, the expert consensus does not recommend the use of olfaction as a RAPA when patients complain of a COVID-19 PACS history (level II, grade B). Many PACS patients continue to complain of olfactory disorders 1 year after the COVID-19 onset, however, to date, not enough high-quality studies report a complete recovery amongst those undergoing either subjective testing or psychophysical olfactory testing (mainly on identification).

#### 3.2.2. Consensus recommendation: graphical marker-related remote digital assessments for preclinical Alzheimer's disease

Only one review (Deer et al., 2021) reported a study with 4% of hand muscle weakness in PACS patients that directly involve graphical markers. However, many indirect symptoms were reported (Ahmad et al., 2021; Davis et al., 2021; Deer et al., 2021; Silva Andrade et al., 2021; Jamoulle et al., 2022; Pinzon et al., 2022; Ser et al., 2022) with potential impacts on graphical capacities such as pins and needles and numbness in hand (2%) (Ahmad et al., 2021), and fatigue or muscle weakness (63%) (Ahmad et al., 2021). From a physiological point of view, even if cutaneous sensitivity and conductance parameters were significantly measured as abnormal in PACS patients reporting autonomic complaints, no nerve conduction abnormalities were reported in the literature (Ser et al., 2022). In parallel, many symptoms were reported that could indirectly influence graphical markers such as abnormal exteroceptive sensation (13.8%), abnormality of movements (2%), and dysmetria (2.8%) (Deer et al., 2021); muscle spasms (22%), tremors (28%), vibrating sensations (18%), and tactile hallucinations (3.1%) (Davis et al., 2021); or skeletomuscular global impairment (Davis et al., 2021; Deer et al., 2021; Silva Andrade et al., 2021) with pain (Pinzon et al., 2022) (27.8%), paresthesia (Pinzon et al., 2022) (33.3%), or movement disorders (Pinzon et al., 2022) (3.6%). In a cohort study, Jamoulle et al. (2022) reported the case of a man infected with COVID-19 three times who developed anosmia, dysgeusia, severe cognitive and memory problems, and alteration of cerebral perfusion on SPEC-CT: he complained about paraesthesia in his fingertips, lateral hand tremors, and instances when his hands opened by themselves when doing specific tasks, which caused him to spontaneously drop objects he was carrying.

Considering the above discussion, the expert consensus is to continue using graphical markers in RAPA studies. Rarely did the research report hand skeletomuscular impairments that could lead to graphical marker abnormal results. No PACS studies reported kinetic results and no nerve conductivity abnormalities were reported in this review (level II, grade B).

#### 3.2.3. Consensus recommendation: eye-tracking-related remote digital assessments for preclinical Alzheimer's disease

Three studies (Deer et al., 2021; Silva Andrade et al., 2021; Pinzon et al., 2022) spotted ocular complications in PACS patients described as visual impairments, arterial thrombosis, or ophthalmoplegia, but only one review reported a case of a 28-year-old man with thalassemia minor complaining of gaze-evoked nystagmus and intermittent diplopia on lateral gaze that persisted 10 days after hospital discharge. Five other studies (Ahmad et al., 2021; Davis et al., 2021; Deer et al., 2021; Fernández-de-Las-Peñas et al., 2021; Michelutti et al., 2022) reported indirect potential impairment of eye-tracking tests results mainly the 6 first months after COVID-19 onset: “eyes problems” 79 ± 17 days after COVID-19 onset (Ahmad et al., 2021); visual loss 10–14 weeks after COVID-19 onset (Ahmad et al., 2021); blindness in one study (Deer et al., 2021); blurred vision (9.7–35.7% 7 months after COVID-19 onset) (Davis et al., 2021; Deer et al., 2021); conjunctivitis (8.9%) (Deer et al., 2021); diplopia (6.9%) (Deer et al., 2021) and keratoconjunctivitis (28.6%) (Deer et al., 2021); visual hallucinations (10.4%) (Davis et al., 2021) or finally persistence of visual disturbance in 3.3–8% (Michelutti et al., 2022).

Considering the above discussion, the expert consensus is to continue using eye-tracking markers in RAPA studies as few studies report direct vision and/or oculomotor impairment that could lead to abnormal eye-tracking assessment results (level II, grade B).

#### 3.2.4. Consensus recommendation: central hearing-related remote digital assessments for preclinical Alzheimer's disease

In this review, 4 studies (Deer et al., 2021; Parker et al., 2021; Silva Andrade et al., 2021; Pinzon et al., 2022) reported direct persistent hearing impairment (Silva Andrade et al., 2021) with 6.6% (Deer et al., 2021) to 15% (Parker et al., 2021) persistent sensorineural hearing loss (before 6 months) (Pinzon et al., 2022) without precision on the follow-up and recovery. In parallel, indirect persistent hearing impairments were related to persistent tinnitus and earache (2.5 to 3.6%) (Ahmad et al., 2021; Deer et al., 2021), hyperacusis (34.7%), pulsatile tinnitus (19%), or tinnitus (29%) (Deer et al., 2021; Pinzon et al., 2022). In a systematic review (De Luca et al., 2022a) reported in this work, the authors reported a controlled study of 27 PACS patients (Vs 20 control) 3.81 ± 2.11 months after COVID-19 onset where speech audiometry showed small but significant impairment in PACS correlated in auditory brainstem response to a lengthening of waves III-V interpeak latencies. However, in the same work (De Luca et al., 2022a) the authors report other studies that failed to show any differences in vestibular or cochlear, even retro cochlear, function (auditory brainstem responses).

Considering the above discussion, the expert consensus is to still use central hearing markers in RAPA studies as few works report the possibility that PACS may cause damage to the hearing system and so lead to long-lasting abnormal central hearing results (level II, grade B).

#### 3.2.5. Consensus recommendation: vocal and speech test-related remote digital assessments for preclinical Alzheimer's disease

Three studies (Ahmad et al., 2021; Davis et al., 2021; Deer et al., 2021) reported indirect impairment of speech and language in 49% of PACS patients. Seven months after the onset of COVID-19, 22 % reported difficulty speaking, 47% reported difficulties finding the right word, 30% had difficulties communicating verbally, 17% were slurring words, and 9% reported speaking unrecognizable words. Problems swallowing were reported in a 39 PACS patient cohort study, although no specifics were given (Ahmad et al., 2021). Different types of aphasia were reported in an 81-cohort systematic review (Deer et al., 2021) [anomic in one study (46.3%), bilingual in one study (28.9%), expressive in one study (22.2%), receptive in one study (23.8%)] with the possibility that COVID-19 could lead to vocal and spontaneous speech impairments (flow rate, hesitations). Only 3 studies (Davis et al., 2021; Deer et al., 2021; Silva Andrade et al., 2021) underlined a direct speech impairment with 7 months after COVID-19 onset 38% speech and language issues (Davis et al., 2021) like slurred speech were reported for 15.8% of patients in a review of 59 PACS papers (Deer et al., 2021). In a narrative review of vocal and speech tests (Silva Andrade et al., 2021), the authors reported the case of a 49-year-old woman with COVID-19 infection who exhibited no flu symptoms but suddenly presented speech disorder and left-side hemiparesis related to a couple of small acute cerebral infarctions in the right prerolandic cortex, which is a rare complication of COVID-19. However, no follow-up data were included.

Considering the above discussion, the expert consensus is to continue to use vocal markers in RAPA studies as few studies reported the possibility that COVID-19 infection may cause significant modifications to vocal performances and lead to abnormal vocal assessments results (level II, grade B).

#### 3.2.6. Consensus recommendation: spatial navigation abilities test-related remote digital assessments for preclinical Alzheimer's disease

Only 2 papers (Pinzon et al., 2022) [Bertuccelli et al. (2022)], included in this work, reported PACS patients with symptoms related to spatial navigation ability impairments. In the first paper (Pinzon et al., 2022) authors reported 2.6% of persistent spatial disorientation and/or confusion in a 697 PACS patient cohort of 63 ± 14.4-year-olds on average, 6 months after hospital discharge. In the second paper [Bertuccelli et al. (2022)], authors analyzed MoCA visuo-spatial subitems and reported the mean score of a sample of 29 non-ICU-admitted subjects, 0–3 months after symptoms onset: they revealed impaired spatial navigational functions (2.50 ± 1.34;max score:4). Moreover, in the same review [Bertuccelli et al. (2022)], five other studies assessed visuo-spatial abilities with visual reproduction of the Wechsler Memory Scale, the Rey-Osterrieth Complex Figure, and the Corsi Test, none of which found relevant deficits.

Considering the above discussion, the expert consensus is to keep using spatial navigation RAPAs, as few studies in the literature are controversial. This indicates that there is a weak possibility that COVID-19 infection may cause significant modifications to the performance of spatial navigation (level II, grade B).

## 4. Discussion

This study of the literature has aimed to assess the potentially impacted RAPAs in a PACS situation, and to assess the expert consensus or recommendations when considering PACS medical history for each RAPA. The recommendations, based on expert consensus, are directed by physicians, neuroscientists, clinicians, or students working on AD early diagnosis and indicate the importance of keeping in mind COVID-19′s potential influence on results. Clearly, PACS-reporting patients may not be able to be screened for AD efficiently and special attention must be paid to the choice of which early markers to use in making assessments.

A total of 20 studies met our inclusion criteria. The main finding concerns the presence of olfaction persistent impairments, which might seriously affect the validity of olfactory screening for neurodegenerative diseases. This scoping review raises two questions: First, the similarities between PACS and AD. The second is relative to the impact of PACS on RAPA targets, which could potentially hinder any AD screening due to the biased results produced by PACS outcomes.

Since the beginning of the pandemic, many authors have drawn attention to the similarity and connection between COVID-19 and AD. While the cerebral invasiveness of COVID-19 is still being investigated, the inflammatory consequences of COVID-19 on the brain have been demonstrated. Furthermore, many arguments link COVID-19 infection and AD (Verkhratsky et al., 2020; Mahalaxmi et al., 2021; Chen et al., 2022; Li et al., 2022a). The trans-endothelial mechanism is highly discussed as the main way of systemically spreading COVID-19 (Chen et al., 2022). However, olfactory neuroepithelium and olfactory neurons could be an alternative means of transmission. (Meinhardt et al., 2021; Ziuzia-Januszewska and Januszewski, 2022). Viruses, like HSV or EBV infection or reactivation, might play an important role in AD genesis (Ou et al., 2020) and could be self-sustained, for example by the fourth isoform of apolipoprotein E genotype (APOE4). APOE4 is a well-known AD risk factor and has been reported to facilitate HSV1 reactivation in the brain through many events such as immunosuppression, peripheral infection, or inflammation (Abate et al., 2020). Many authors speculate on long-lasting inflammation in PACS patients with astrocytes and microglia brain activation polarized in a facilitating way (M1 phenotype) of ß-amyloid and Tau phosphorylation levels increase (Abate et al., 2020; Chen et al., 2022). Moreover, APOE4 may facilitate the infectivity of COVID-19 by regulating intracellular levels of cholesterol and increasing the S-protein binding to ACE2 (Chen et al., 2022) but its PACS role and staying power are debated in clinical trials (Tavares-Júnior et al., 2022). Wide ACE2 binding during COVID-19 infection could downregulate the ACE2 receptor (Chen et al., 2022) for a while, which has been reported to be decreased in post-mortem brain tissue of AD patients, and inversely is correlated to ß-amyloid levels and Tau phosphorylation (Kehoe et al., 2016). Finally, ß-amyloid, a peptide with antimicrobial properties, may be an innate immune system actor (Soscia et al., 2010) but could be theoretically and ironically over-produced in PACS patients. Given that ~659 million people have so far been infected by COVID-19, more follow-up with PACS patients and more powerful, high-quality studies need to be undertaken.

### 4.1. Olfaction RAPAs are no longer recommended

RAPA target assessments could interfere with PACS outcomes, and as such, this type of assessment may complicate potential AD early screenings. As the experts' conclusions (Table 3) underline, olfaction could be an early AD marker. An identification impairment without any other etiology is an early symptom of phosphorylated Tau protein neurofibrillary tangles (NFTs) and ß-amyloid plaques accretion in olfactory bulbs and entorhinal cortex (De Luca et al., 2022b), which is the main cortex gate between a smell and its memory and one of the first brain-impaired regions in early AD (Saramago and Franceschi, 2021), with the hippocamp and amygdala. Olfactory Identification subdimension is impaired in PACS and reflects the olfactory subjective (visual analogic scale) and patient quality of life impairment (Vandersteen et al., 2021). Almost 2 years (Lechien et al., 2023) after the onset of COVID-19, 29.8% of PACS patients still complain of olfactory disorders (0.6% of hyposmic and 2.3% anosmic on identification psychophysical test results) with 13.4% of parosmia. Parosmias are one of the main olfactory-persistent symptoms of PACS patients (Davis et al., 2021) and are only predictive of a threshold impairment (Menzel et al., 2022). Therefore, just as we see in older patients, there will be a global olfactory score improvement but not an olfactory identification score improvement (Gary et al., 2023; Lechien et al., 2023). Our results indicate the presence of more hyposmia, anosmia, and parosmia from the time of COVID-19 onset. As reported in this review, the lack of psychophysical olfactory tests in the included studies (mainly subjective assessments) seems to overestimate, from a quantitative perspective, olfactory disorders for more than 40% of patients (Nørgaard and Fjaeldstad, 2021). Dysgeusia was frequently reported in PACS in similar proportion to dysosmia. As retro-olfaction is often confused with taste in 50% of people (Nørgaard and Fjaeldstad, 2021), clinicians have to pay attention to “dysgeusia” as it could be an olfaction impairment because gustatory functions are rarely impacted during COVID-19 onset (Hintschich et al., 2020) and when they are, they are short-lived (Chiesa-Estomba et al., 2020). Lack of psychophysical olfactory testing, frequent long-lasting dysosmia, and risk of dysosmia misdiagnoses because of false dysgeusia in the PACS literature prevent us from specifying with precision PACS-persistent olfactory disorders and features and therefore potential long-lasting identification impairment. This is why experts recommend, for the moment, not to trust olfactory identification impairments for RAPA in PACS patients until more high-quality studies are published. Only then will researchers be able to assert if there is complete olfaction recovery.

### 4.2. Graphical marker RAPAs are still recommended

As the experts conclude, graphical markers are widely studied in RAPA, as AD patients report a decline in fine motor control, coordination, and writing or drawing impairments that compromise daily life activities (Yan et al., 2008). AD hand movements become slower, less fluid, and less consistent due to reduced precision in wrist and finger positioning (Impedovo and Pirlo, 2018). Handwriting pressure decreases in patients with AD when cognitive tasks are performed (Plonka et al., 2021)allowing physicians to differentiate them from healthy controls. Moreover the increase of writing time between two strokes [known as pen-up time (Alfalahi et al., 2022)] is reported to be a key discriminator (Delazer et al., 2021) between AD and Mild Cognitive Impairment (MCI) patients compared to healthy individuals when performing tasks that involve visuospatial construction, cognitive writing, or the Clock Drawing Test (Werner et al., 2006; Müller et al., 2017a,b). For MCI and AD screening, pooled sensitivity and specificity of kinetics are respectively 0,85 and 0,82 in a Scientific Report study (Alfalahi et al., 2022) and allow, specifically for drawing tasks (spiral, crossed pentagons, 3D house, clock drawing test), a high specificity to screen MCI or AD patients (Garre-Olmo et al., 2017). In this research, few symptoms were reported in PACS patients that interfere with direct kinetic assessments [such as tremors and hand muscle weakness Deer et al., 2021] or could influence indirectly the way the patient writes or draws [such as vibrating sensations, tactile hallucinations, abnormal exteroceptive sensation or paresthesia Davis et al., 2021; Deer et al., 2021]. The literature reports upper extremity plexopathy (Li et al., 2022b; Michaelson et al., 2022) in severe COVID-19 (requiring mechanical ventilation) between 1 to 3 months after infection onset. However, the responsibility of the prone position is still debated (King-Robson et al., 2022) and no recovery, long follow-up, or specific kinetic studies have yet been performed on these neurological PACS patients. This explains the important loss in specificity that is evaluated by experts (Table 3), who nevertheless recommend still using this RAPA.

### 4.3. Speech RAPAs are still recommended

For over a decade, many authors have worked on AD -connected speech assessments (Boschi et al., 2017), as confirmed by experts' interest in this RAPA, especially since the development of computer-assisted voice analysis. These speech and voice assessments have focused on the lexico-semantic and discourse-pragmatic aspects, which account for around 80 and 77.5% of the actual research, respectively (Boschi et al., 2017). The syntactic, phonetic and phonemic, and finally morphological aspects comprise, respectively, 57.5, 55, and 35% of current studies (Boschi et al., 2017). In AD patients phonetic and phonological errors have been reported, as well as a low speech rate and increase in hesitations (Hoffmann et al., 2010; Sajjadi et al., 2012), lexico-semantic errors, word findings difficulties (Forbes-McKay et al., 2013), and a greater number of closed class (Drummond et al., 2015) and high-frequency words (Kavé and Levy, 2003). In this review, frequent [49% Davis et al., 2021] impairments of speech were reported in PACS patients with imprecise speech and language issues that could be linked more to lexico-semantic, phonetic, and phonological features. In PACS patients, general (Ahmad et al., 2021) and verbal communication difficulties or slurring words were reported (Davis et al., 2021) as did cross lexical and semantic RAPA or different types of aphasia (Deer et al., 2021). However, few studies reported speech-specific PACS in the 6 months after the onset of COVID-19. Although remote European (semi) automated speech analysis projects are being carried out, this sort of specific speech/acoustic measures are rarely investigated (Boschi et al., 2017) in AD and never recorded in PACS. Because of these short-lasting voice PACS and without acoustic persistent issues, experts recommend continuing using speech RAPA.

### 4.4. Eye tracking, visual abilities, and central hearing RAPAs are still recommended

Finally, these three RAPAs were not suggested as significatively relevant by experts. Eye-tracking as RAPA is based on eye saccades and fixation recording during specific tasks (reading, cognitive, or memory test) through devices embedded-cameras (laptop, tablet, phone). Eye-tracking has been validated in AD and MCI screening 10 years ago (Peltsch et al., 2014; Seligman and Giovannetti, 2015) with a cognitive impairment diagnosis sensitivity and specificity of respectively 0,75 and 0,73 in a recent systematic review and meta-analysis (Liu et al., 2021). In this review, only one case was reported (Deer et al., 2021) with a saccades-modifying condition. All other eye-tracking-related studies pointed to potential fixation difficulties (Ahmad et al., 2021; Davis et al., 2021; Deer et al., 2021; Fernández-de-Las-Peñas et al., 2021; Michelutti et al., 2022) in PACS patients but these were presumed to be curable for some items (Deer et al., 2021) [conjunctivitis (8.9%), keratoconjunctivitis (28.6%)]. However, good vision is mandatory to be able to use new spatial navigation assessments in addition to good visuospatial cognitive functions. Even if VR computer-generated environments were used to assess spatial navigation, RAPAs (Öhman et al., 2021) could destabilize AD older patients. PACS was younger, and zero to few spatial navigation abilities impairments were reported. Eye-tracking has been validated in combination with virtual reality (VR) simulation (Davis, 2021) as a RAPA, but under some conditions, there is a mismatch between the use of contemporary technologies and AD/control patients age (VR induced nausea, the inability to calibrate a device, or understand the instructions). Finally, persistent visual disturbances were reported in 3.3% (*n* = 5/151) to 8% (*n* = 5/62) of a 213 PACS cohort observational study, 3 months after the onset of COVID-19 (Michelutti et al., 2022).

The last RAPA is central hearing, which is less relevant for experts. Central hearing RAPA includes auditory temporal processing, dichotic tests, monaural low-redundancy speech tests, and auditory discrimination and memory tests (Tarawneh et al., 2022). These all depend on possessing efficient sensorineural hearing, which was reported as impaired in 4 short follow-up studies (Deer et al., 2021; Parker et al., 2021; Silva Andrade et al., 2021; Pinzon et al., 2022) and in up to 15% of PACS patients. Moreover, central hearing assessments depend on cognitive, memory, and attention abilities which could be widely impaired in respectively 70–90%, 70%, and 50–90% of a 3762 PACS patients' observational cohort. Approximately 7 months after the onset of COVID-19, (Davis et al., 2021), cognitive impairments are one of the three most frequently reported symptoms. One year after COVID-19 onset, in a non-included review, memory loss and attention abilities were still impaired in 19% and 18% of an 8591 patient PACS cohort (Han et al., 2022). Tinnitus and hyperacusis were reported in less than 30% of PACS patients, which could add a negative effect on hearing. Despite persistent hearing disorders reported in this review, no study reported central auditory tests on PACS patients justifying experts' recommendation to carry on using this RAPA.

This study has several limits. PACS has been gradually defined since the pandemic started until 6 October 2021 when a World Health Organization DELPHI consensus provided a clinical definition of PACS for adults and 16 February 2023 for children and adolescents. This evolving definition explains the extreme variability of assessment times in every review reported in this work, running from 2 to 52 weeks, and mainly in the first 30 weeks after the onset of COVID-19 (Table 1). This variability in definition could have contributed to overestimating PACS sustainability and as such, the recommendations that were made at the time. Moreover, many reviews can be classified as low or critically low quality because most of them did not provide a meta-analysis of their data, which can result in heterogeneity and a risk of biased assessments. Four of the six cohort observational studies received more than 75% quality score, a full score indicating a perfect level of quality. For the main concerns, bias was methodological. Observational studies (level 2 HAS scientific evidence) only allowed for a presumption of scientific quality compared to level 1 studies which were, mostly here, of low quality. Finally, COVID-19 papers and as such PACS ones, have been part of a larger phenomenon which consists in an increase of COVID-19-related publication numbers, a decrease in review time, and finally, a decline in methodological quality (Jung et al., 2021).

## 5. Conclusion

This work highlights the value of using RAPAs to screen preclinical AD, including PACS patient population. However, the stratification of RAPAs is essential in the post-COVID-19 period. Graphical, SPEECH, eye-tracking, central hearing, and spatial navigation abilities are still usable without any concern, but olfactory function may be altered by PACS and should be avoided in a preclinical AD screening assessment. This consensus statement will require an update after a few years to guarantee that treatments and recommendations continue to be supported by the latest evidence. More longitudinal studies are required to provide more evidence for the future of RAPA target modifications in PACS patients.

## Data availability statement

The raw data supporting the conclusions of this article will be made available by the authors, without undue reservation.

## Author contributions

AG led the supervision of this project. CV and AP organized the database and the first draft of the manuscript. VM and AG performed the entire consensus recommendations in which all authors took part. KS performed the extensive English editing. All authors contributed to the conception and design of the study, research study, manuscript revision, read, and approved the submitted version.

## Footnotes

^1^ https://apps.who.int/iris/rest/bitstreams/1376291/retrieve

^2^ https://www.who.int/publications/i/item/WHO-2019-nCoV-clinical-2021-2
