# Supplementary material for: Alzheimer's early detection in post-acute COVID-19 syndrome: a systematic review and expert consensus on preclinical assessments
Source: Front Aging Neurosci. 2023 Jun 21;15:1206123. doi: 10.3389/fnagi.2023.1206123 (PMC10320294; doi:10.3389/fnagi.2023.1206123)
Supplement: Supplementary file 1 [file Data_Sheet_1.docx]

Supplementary Material

Detailed Search Request Strategy

Clair Vandersteen1*†, Alexandra Plonka†, Valeria Manera, Kim Sawchuk, Constance Lafontaine, Kevin Galery, Olivier Rouaud, Nouha Bengaied, Cyrille Launay, Olivier Guérin, Philippe Robert, Gilles Allali, Olivier Beauchet, Auriane Gros

***Correspondence:** Corresponding Author: [vandersteen.c@chu-nice.fr](mailto:vandersteen.c@chu-nice.fr)

**Supplementary Figure**

**Supplementary Figure 1.** Detailed Search Request Strategy for identifying papers related to Remote digital Assessments for Preclinical Alzheimer’s disease potentially impacted in post-acute COVID-19 syndrome patients.
