# Supplementary material for: Alzheimer's early detection in post-acute COVID-19 syndrome: a systematic review and expert consensus on preclinical assessments
Source: Front Aging Neurosci. 2023 Jun 21;15:1206123. doi: 10.3389/fnagi.2023.1206123 (PMC10320294; doi:10.3389/fnagi.2023.1206123)
Supplement: Supplementary file 2 [file Data_Sheet_2.docx]

Supplementary Material

Complete Process of Remote digital Alzheimer’s Disease preclinical assessments’ identification

Clair Vandersteen^1*^†, Alexandra Plonka†, Valeria Manera, Kim Sawchuk, Constance Lafontaine, Kevin Galery, Olivier Rouaud, Nouha Bengaied, Cyrille Launay, Olivier Guérin, Philippe Robert, Gilles Allali, Olivier Beauchet, Auriane Gros

***Correspondence:** Corresponding Author: [vandersteen.c@chu-nice.fr](mailto:vandersteen.c@chu-nice.fr)

# Introduction

The first step of this review was to identify potential promising remote digital AD preclinical assessments (RAPAs). RAPAs are different from common preclinical AZ biomarkers in that they must be independent of material and human factors and significantly efficient in the preclinical phase. They should be fully digitized and easily understandable by anyone with an internet connection, meaning that the local results (i.e. before web sending) must be robust and less dependent on technical factors. Moreover, the required human expertise and time-consuming effect should be low so that the test(s) can be performed quickly by non-medical staff or even by the patient themselves.

# Method

To meet these requirements, PET/TAU-PET, lumbar puncture, MRI, biological tests, and cognitive assessments were excluded. To focus on evaluated and validated RAPAs, only English-language reviews or systematic reviews were included in this preliminary study.

A quick but systematic review was undertaken searching on PubMed, the 15/11/2022 in the 5 last year’s literature, papers related to these keywords : ("alzheimer" OR "alzheimer disease") AND ("biomarker" OR "biomarkers" OR "markers") AND ("early diagnosis" OR "early detection" OR "preclinical" OR "digital assessment") NOT ("biological" OR "imaging" OR "PET" OR "CSF" OR "lumbar puncture" OR "blood" OR "Parkinson disease").

# Results and discussion

Two reviewers (CV/AP) reviewed the titles and abstracts of 97 studies. The first RAPA identified was olfactory dysfunction(Kotecha et al., 2018), which occurs not only in the initial stages of AD (Mild Cognitive Impairment - MCI) but also prior to preclinical stages. Meta-analyses have shown that the identification olfaction subdimension is significantly impacted in MCI and proven AZ(Kotecha et al., 2018). Identification olfactory-related tests are recognized as screening or prognostic targets for AZ onset(Payne et al., 2022). Despite the lack of histologic human studies data, olfactory identification impairment is thought to be linked to TAU-neurofibrillary and amyloid-ß deposition in the olfactory bulb, entorhinal cortex, and hippocampal cortex, leading to the inability to store and remember smell memories(Roberts et al., 2016). Olfactory tests are easy to administer, inexpensive (commercialized), and non-invasive.

The second RAPA concerns fine motor decline and impairment in writing or drawing related tasks. A deterioration in fine motor skills and coordination occurs(Yan et al., 2008) in AD patients, leading to slower, less smooth, less coordinated, and less consistent handwriting movements due to reduced ability in wrist and finger fine positioning(Impedovo et Pirlo, 2018). Moreover, the "in-air" time between two strokes, with a tablet pencil or directly on the screen (keystrokes(Alfalahi et al., 2022)), also known as flight time, is specifically discriminant for AD and MCI from healthy controls with visuospatial construction tasks, cognitive writing tasks, or the Clock Drawing Test(Werner et al., 2006; Müller et al., 2017a, 2017b). These assessments could easily be done on an e-tablet or phone.

The third RAPA is related to the voice and a new connected speech recording and analyzing tool(Öhman et al., 2021). AD-related speech and voice impairments include phonetic and phonological, lexico-semantic, morpho-syntactic, discourse, and pragmatic level impairments(Boschi et al., 2017). AD patients seem to have significantly less idea density (lexico-semantic features) and less information content, index of discourse effectiveness, and information units efficiency (discourse and pragmatic features) than MCI(Boschi et al., 2017). Speech production is currently recorded during spontaneous speech, verbal fluency, or picture describing tasks. A common microphone connected to computer software allows analyzing speech, sentence and grammatical complexity, variability of words, and finally speaking flow(Mueller et al., 2017). It could be hard to identify abnormalities using conventional neuropsychological tests, but some authors underlined little correlation between preclinical AD patients and subtle speech changes(Verfaillie et al., 2019).

The fourth identified RAPA is eye-tracking impairment to screen abnormal movement behaviors (saccades, fixation). Mobile versions (on iPad devices) are already developed for mild and severe cognitive impairment (Accuracy of 76%) with the same efficiency as commercial eye-tracking hardware(Haque et al., 2021). Many authors (Bott et al., 2020; Gills et al., 2021; Tadokoro et al., 2021) have already used these devices to identify progressive cognitive decline with significant effectiveness.

The fifth RAPA is central hearing impairment. Hearing loss is a well-known 8% modifiable but contributing factor to dementia (Lin et al., 2011; Livingston et al., 2020). Temporal auditory processing is associated with cognitive impairment, as central auditory processing assessments (adaptive tests of temporal response (ATTR), time-compressed speech test (TCS), Dichotic Digits Test (DDT), Dichotic Sentence Identification (DSI), Speech in Noise (SPIN), and Synthetic Sentence Identification-Ipsilateral Competing Message (SSI-ICM)) have been reported to be impaired in mild cognitive impairment patients compared to a healthy control group(Tarawneh et al., 2022). Some authors proposed a systematic central auditory dysfunction screening in at-risk populations to identify early AD in a low-cost way(Swords et al., 2018), specifically dichotic tests(Gates et al., 2008; Idrizbegovic et al., 2011; Häggström et al., 2020).

The last RAPA is virtual reality and spatial navigation abilities. With dedicated cognitive virtual reality-based platform development on nonspecific equipment (i-Devices, for example(Moodley et al., 2015; Ritchie et al., 2018)), accessibility and use of these tools are growing. Recently, authors reported that an entorhinal cortex-based test of virtual reality navigation was able to significantly identify early MCI patients. Serious games are probably part of the future of virtual reality assessments, as reported by authors who used a mobile game (Sea Hero Quest) to underline that 3D navigation ability assessed in that game was able to differentiate high-risk of AD healthy people based on genetic (APOE) and demographic risk factors.

Given the multifactorial nature of AD (both neurologically and clinically), it is unlikely to be possible to produce a single biomarker of sufficient value for clinical diagnosis at this point. Efforts should be directed toward standardizing a panel of early biomarkers (including probably some of those mentioned above) capable of diagnosing MCI with sufficient sensitivity and specificity.

# References

Alfalahi, H., Khandoker, A. H., Chowdhury, N., Iakovakis, D., Dias, S. B., Chaudhuri, K. R., et al. (2022). Diagnostic accuracy of keystroke dynamics as digital biomarkers for fine motor decline in neuropsychiatric disorders: a systematic review and meta-analysis. *Sci. Rep.* 12, 1‑24. doi: 10.1038/s41598-022-11865-7.

Boschi, V., Catricalà, E., Consonni, M., Chesi, C., Moro, A., et Cappa, S. F. (2017). Connected Speech in Neurodegenerative Language Disorders: A Review. *Front. Psychol.* 8, 269. doi: 10.3389/fpsyg.2017.00269.

Bott, N. T., Madero, E. N., Glenn, J. M., Lange, A. R., Anderson, J. J., Newton, D. O., et al. (2020). Device-Embedded Cameras for Eye Tracking-Based Cognitive Assessment: Implications for Teleneuropsychology. *Telemed. J. E. Health.* 26, 477‑481. doi: 10.1089/tmj.2019.0039.

Gates, G. A., Anderson, M. L., Feeney, M. P., McCurry, S. M., et Larson, E. B. (2008). Central auditory dysfunction in older persons with memory impairment or Alzheimer dementia. *Arch. Otolaryngol. Head. Neck Surg.* 134, 771‑7. doi: 10.1001/archotol.134.7.771.

Gills, J. L., Bott, N. T., Madero, E. N., Glenn, J. M., et Gray, M. (2021). A short digital eye-tracking assessment predicts cognitive status among adults. *GeroScience* 43, 297‑308. doi: 10.1007/s11357-020-00254-5.

Häggström, J., Hederstierna, C., Rosenhall, U., Östberg, P., et Idrizbegovic, E. (2020). Prognostic Value of a Test of Central Auditory Function in Conversion from Mild Cognitive Impairment to Dementia. *Audiol. Neurotol.* 25, 276‑282. doi: 10.1159/000506621.

Haque, R. U., Pongos, A. L., Manzanares, C. M., Lah, J. J., Levey, A. I., et Clifford, G. D. (2021). Deep Convolutional Neural Networks and Transfer Learning for Measuring Cognitive Impairment Using Eye-Tracking in a Distributed Tablet-Based Environment. *IEEE Trans. Biomed. Eng.* 68, 11‑18. doi: 10.1109/TBME.2020.2990734.

Idrizbegovic, E., Hederstierna, C., Dahlquist, M., Nordström, C. K., Jelic, V., et Rosenhall, U. (2011). Central auditory function in early Alzheimer’s disease and in mild cognitive impairment. *Age Ageing* 40, 249‑254. doi: 10.1093/ageing/afq168.

Impedovo, D., et Pirlo, G. (2018). Dynamic Handwriting Analysis for the Assessment of Neurodegenerative Diseases: A Pattern Recognition Perspective. *IEEE Rev. Biomed. Eng.* 12, 209‑220. doi: 10.1109/RBME.2018.2840679.

Kotecha, A. M., Corrêa, A. D. C., Fisher, K. M., et Rushworth, J. V (2018). Olfactory Dysfunction as a Global Biomarker for Sniffing out Alzheimer’s Disease: A Meta-Analysis. *Biosensors* 8, 41. doi: 10.3390/bios8020041.

Lin, F. R., Metter, E. J., O’Brien, R. J., Resnick, S. M., Zonderman, A. B., et Ferrucci, L. (2011). Hearing loss and incident dementia. *Arch. Neurol.* 68, 214‑20. doi: 10.1001/archneurol.2010.362.

Livingston, G., Huntley, J., Sommerlad, A., Ames, D., Ballard, C., Banerjee, S., et al. (2020). Dementia prevention, intervention, and care: 2020 report of the Lancet Commission. *Lancet* 396, 413‑446. doi: 10.1016/S0140-6736(20)30367-6.

Moodley, K., Minati, L., Contarino, V., Prioni, S., Wood, R., Cooper, R., et al. (2015). Diagnostic differentiation of mild cognitive impairment due to Alzheimer’s disease using a hippocampus-dependent test of spatial memory. *Hippocampus* 25, 939‑51. doi: 10.1002/hipo.22417.

Mueller, K. D., Koscik, R. L., Hermann, B. P., Johnson, S. C., et Turkstra, L. S. (2017). Declines in Connected Language Are Associated with Very Early Mild Cognitive Impairment: Results from the Wisconsin Registry for Alzheimer’s Prevention. *Front. Aging Neurosci.* 9, 437. doi: 10.3389/fnagi.2017.00437.

Müller, S., Preische, O., Heymann, P., Elbing, U., et Laske, C. (2017a). Diagnostic Value of a Tablet-Based Drawing Task for Discrimination of Patients in the Early Course of Alzheimer’s Disease from Healthy Individuals. *J. Alzheimer’s Dis.* 55, 1463‑1469. doi: 10.3233/JAD-160921.

Müller, S., Preische, O., Heymann, P., Elbing, U., et Laske, C. (2017b). Increased Diagnostic Accuracy of Digital vs. Conventional Clock Drawing Test for Discrimination of Patients in the Early Course of Alzheimer’s Disease from Cognitively Healthy Individuals. *Front. Aging Neurosci.* 9, 101. doi: 10.3389/fnagi.2017.00101.

Öhman, F., Hassenstab, J., Berron, D., Schöll, M., et Papp, K. V. (2021). Current advances in digital cognitive assessment for preclinical Alzheimer’s disease. *Alzheimer’s Dement. Diagnosis, Assess. Dis. Monit.* 13, 1‑19. doi: 10.1002/dad2.12217.

Payne, M., Manera, V., Robert, P., Vandersteen, C., Beauchet, O., Galery, K., et al. (2022). Olfactory identification disorders due to Alzheimer’s disease: A new test from France to Quebec. *PLoS One* 17, 1‑13. doi: 10.1371/journal.pone.0265764.

Ritchie, K., Carrière, I., Howett, D., Su, L., Hornberger, M., O’Brien, J. T., et al. (2018). Allocentric and Egocentric Spatial Processing in Middle-Aged Adults at High Risk of Late-Onset Alzheimer’s Disease: The PREVENT Dementia Study. *J. Alzheimers. Dis.* 65, 885‑896. doi: 10.3233/JAD-180432.

Roberts, R. O., Christianson, T. J. H., Kremers, W. K., Mielke, M. M., Machulda, M. M., Vassilaki, M., et al. (2016). Association Between Olfactory Dysfunction and Amnestic Mild Cognitive Impairment and Alzheimer Disease Dementia. *JAMA Neurol.* 73, 93‑101. doi: 10.1001/jamaneurol.2015.2952.

Swords, G. M., Nguyen, L. T., Mudar, R. A., et Llano, D. A. (2018). Auditory system dysfunction in Alzheimer disease and its prodromal states: A review. *Ageing Res. Rev.* 44, 49‑59. doi: 10.1016/j.arr.2018.04.001.

Tadokoro, K., Yamashita, T., Fukui, Y., Nomura, E., Ohta, Y., Ueno, S., et al. (2021). Early detection of cognitive decline in mild cognitive impairment and Alzheimer’s disease with a novel eye tracking test. *J. Neurol. Sci.* 427, 117529. doi: 10.1016/j.jns.2021.117529.

Tarawneh, H. Y., Menegola, H. K., Peou, A., Tarawneh, H., et Jayakody, D. M. P. (2022). Central Auditory Functions of Alzheimer’s Disease and Its Preclinical Stages: A Systematic Review and Meta-Analysis. *Cells* 11, 1007. doi: 10.3390/cells11061007.

Verfaillie, S. C. J., Witteman, J., Slot, R. E. R., Pruis, I. J., Vermaat, L. E. W., Prins, N. D., et al. (2019). High amyloid burden is associated with fewer specific words during spontaneous speech in individuals with subjective cognitive decline. *Neuropsychologia* 131, 184‑192. doi: 10.1016/j.neuropsychologia.2019.05.006.

Werner, P., Rosenblum, S., Bar-On, G., Heinik, J., et Korczyn, A. (2006). Handwriting process variables discriminating mild Alzheimer’s disease and mild cognitive impairment. *J. Gerontol. B. Psychol. Sci. Soc. Sci.* 61, P228-36. doi: 10.1093/geronb/61.4.p228.

Yan, J. H., Rountree, S., Massman, P., Doody, R. S., et Li, H. (2008). Alzheimer’s disease and mild cognitive impairment deteriorate fine movement control. *J. Psychiatr. Res.* 42, 1203‑1212. doi: 10.1016/j.jpsychires.2008.01.006.
