# Supplementary material for: Alzheimer's early detection in post-acute COVID-19 syndrome: a systematic review and expert consensus on preclinical assessments
Source: Front Aging Neurosci. 2023 Jun 21;15:1206123. doi: 10.3389/fnagi.2023.1206123 (PMC10320294; doi:10.3389/fnagi.2023.1206123)
Supplement: Supplementary file 3 [file Data_Sheet_3.docx]

Supplementary Material

Supplementary Tables

Clair Vandersteen^1*^†, Alexandra Plonka†, Valeria Manera, Kim Sawchuk, Constance Lafontaine, Kevin Galery, Olivier Rouaud, Nouha Bengaied, Cyrille Launay, Olivier Guérin, Philippe Robert, Gilles Allali, Olivier Beauchet, Auriane Gros

***Correspondence:** Corresponding Author: [vandersteen.c@chu-nice.fr](mailto:vandersteen.c@chu-nice.fr)

# Supplementary Tables

|  | **Representativeness** | | Independent variable: exposure, intervention, or risk factor | | **Dependent variable** | | | | | Loss to follow-up | | | **Miscellaneous** | | Optional |  |
| --- | --- | --- | --- | --- | --- | --- | --- | --- | --- | --- | --- | --- | --- | --- | --- | --- |
| **Authors** | Q1 | Q2 | Q3 | Q4 | Q5 | Q6 | Q7 | Q8 | Q9 | Q10 | Q11 | Q12 | Q13 | Q14 | Q15 | **Total score** |
| **(Jamoulle et al., 2022)** | YES | YES | YES | YES | YES | YES | YES | YES | YES | YES | YES | YES | NO | NO | YES | **13/15** |
| **(Davis et al., 2021)** | YES | YES | YES | YES | YES | YES | NO | YES | NO | NO | YES | YES | YES | YES | YES | **12/15** |
| **(Girón Pérez et al., 2022)** | NO | YES | YES | YES | NO | NO | NO | NO | YES | YES | YES | NO | YES | YES | NO | **8/15** |
| **(Michelutti et al., 2022)** | NO | YES | YES | YES | NO | NO | NO | NO | YES | YES | YES | YES | YES | NO | NO | **8/15** |
| **﻿ (Vandersteen et al., 2021)** | NO | YES | YES | YES | YES | NO | YES | NO | YES | YES | YES | YES | YES | YES | YES | **12/15** |
| **(Mendes Paranhos et al., 2022)** | YES | YES | YES | YES | YES | NO | YES | NO | NO | YES | YES | YES | YES | YES | YES | **12/15** |

**Table A**. *Quality assessment of cohort observational studies included in the review using OSQE(Drukker et al., 2021) tool. Questions of this tool are reported below.*

*Question 1 (Q1)- Is the sample optimal for both internal validity and representativeness?*

*Question 2 (Q2) - Is the cohort really one cohort or are there sub-cohorts?*

*Question 3 (Q3) - Is the assessment of the main independent variable valid?*

*Question 4 (Q4) - Is the presence of the independent variable optimal?*

*Question 5 (Q5) - Is the assessment of the dependent variable valid?*

*Question 6 (Q6) - Was exposure unknown to assessor?*

*Question 7 (Q7) - Were subjects where the outcome was present at baseline excluded?*

*Question 8 (Q8) - Is follow-up sufficiently long to assess the outcome?*

*Question 9 (Q9) - Is the outcome assessed continuously?*

*Question 10 (Q10) - Does loss to follow-up likely introduce bias?*

*Question 11 (Q11) - Did the authors use methods to properly deal with missing data?*

*Question 12 (Q12) - Is there a conflict of interest?*

*Question 13 (Q13) - Does the statistical analysis control for the relevant confounders?*

*Question 14 (Q14) - Did the reporting of the results follow a protocol?*

*Question 15 (Q15) - Are effect modifiers analyzed correctly?*

|  | **Representativeness** | | Independent variable: exposure, intervention, or risk factor | | | **Dependent variable** | | | Non-Response | | | | **Comparability / control for confounders** | | Optional |  |
| --- | --- | --- | --- | --- | --- | --- | --- | --- | --- | --- | --- | --- | --- | --- | --- | --- |
| **Authors** | Q1 | Q2 | Q3 | Q4 | Q5 | Q6 | Q7 | Q8 | Q9 | Q10 | Q11 | Q12 | Q13 | Q14 | Q15 | **Total score** |
| **(Ser et al., 2022)** | NO | YES | YES | YES | YES | YES | YES | YES | YES | YES | YES | YES | YES | YES | YES | **14/15** |

**Table B** – *Quality assessment of the only case control study included in the review using OSQE(Drukker et al., 2021) tool.* *Questions of this tool are reported below.*

*Question 1 (Q1)- Is the sample optimal for both internal validity and representativeness?*

*Question 2 (Q2) Is the data collected in one population or are cases and controls selected in different populations?*

*Question 3 (Q3) - Is the assessment of the main independent variable valid?*

*Question 4 (Q4) - Is the presence of the independent variable optimal?*

*Question 5 (Q5) - Is the assessment of the dependent variable valid?*

*Question 6 (Q6) - Is assessment of the dependent variable valid?*

*Question 7 (Q7) - Do controls have a history of the disease?*

*Question 8 (Q8) - Is follow-up sufficiently long to assess the outcome?*

*Question 9 (Q9) - Does non-response likely introduce bias?*

*Question 10 (Q10) - Is non-response similar in cases and controls?*

*Question 11 (Q11) - Did the authors use methods to properly deal with missing data?*

*Question 12 (Q12) - Is there a conflict of interest?*

*Question 13 (Q13) - Does the statistical analysis control for the relevant confounders?*

*Question 14 (Q14) - Did the reporting of the results follow a protocol?*

*Question 15 (Q15) - Are effect modifiers analyzed correctly?*

| Authors | **Q1** | **Q2** | **Q3** | **Q4** | **Q5** | **Q6** | **Q7** | **Q8** | **Q9** | **Q10** | **Q11** | **Q12** | **Q13** | **Q14** | **Q15** | **Q16** | **Overall quality** |
| --- | --- | --- | --- | --- | --- | --- | --- | --- | --- | --- | --- | --- | --- | --- | --- | --- | --- |
| (Ahmad et al., 2021) | YES | NO | YES | NO | YES | YES | NO | YES | NO | YES | NO | NO | NO | NO | NO | YES | **CRITICALLY LOW** |
| (Parker et al., 2021) | YES | NO | YES | NO | NO | NO | NO | ±YES | NO | NO | NO | NO | NO | NO | NO | NO | **CRITICALLY LOW** |
| (Deer et al., 2021) | YES | YES | YES | NO | NO | NO | NO | YES | NO | YES | NO | NO | YES | NO | NO | YES | **CRITICALLY LOW** |
| (Bertuccelli et al., s. d.) | YES | ±YES | YES | ±YES | YES | YES | NO | ±YES | YES | YES | NO | NO | YES | YES | NO | YES | **CRITICALLY LOW** |
| (Dirican et Bal, 2022) | YES | YES | YES | ±YES | YES | YES | NO | YES | YES | YES | YES | YES | YES | YES | YES | YES | **LOW** |
| (De Luca et al., 2022) | YES | NO | NO | ±YES | YES | YES | NO | NO | NO | YES | NO | NO | NO | NO | NO | YES | **CRITICALLY LOW** |
| (Silva Andrade et al., 2021) | YES | ±YES | YES | ±YES | NO | NO | YES | ±YES | YES | YES | NO | YES | NO | YES | NO | YES | **CRITICALLY LOW** |
| (Premraj et al., 2022) | YES | YES | YES | ±YES | YES | YES | YES | YES | YES | NO | YES | YES | YES | YES | YES | YES | **LOW** |
| (Pinzon et al., 2022) | YES | YES | YES | YES | YES | YES | YES | YES | YES | NO | YES | NO | NO | NO | YES | NO | **CRITICALLY LOW** |
| (Malik et al., 2022) | YES | ±YES | YES | ±YES | YES | YES | YES | YES | ±YES | NO | YES | NO | NO | YES | NO | YES | **CRITICALLY LOW** |
| (Xydakis et al., 2021) | YES | NO | NO | NO | NO | NO | NO | NO | NO | NO | NO | NO | NO | YES | NO | YES | **CRITICALLY LOW** |
| (Fernández-de-Las-Peñas et al., 2021) | YES | ±YES | YES | ±YES | YES | YES | ±YES | ±YES | ±YES | NO | YES | YES | NO | YES | YES | YES | **HIGH** |
| (Tan et al., 2022) | YES | YES | YES | ±YES | YES | YES | YES | ±YES | YES | YES | YES | YES | YES | YES | NO | YES | **LOW** |

**Table C** – *Quality assessment of systematic reviews included in the review using AMSTAR2(Shea et al., 2017) tool. Questions of this tool are reported below.*

*Question 1 (Q1) - Did the research questions and inclusion criteria for the review include the components of PICO?*

*Question 2 (Q2) - Did the report of the review contain an explicit statement that the review methods were established prior to the conduct of the review and did the report justify any significant deviations from the protocol?*

*Question 3 (Q3) - Did the review authors explain their selection of the study designs for inclusion in the review?*

*Question 4 (Q4) - Did the review authors use a comprehensive literature search strategy?*

*Question 5 (Q5) - Did the review authors perform study selection in duplicate?*

*Question 6 (Q6) - Did the review authors perform data extraction in duplicate?*

*Question 7 (Q7) - Did the review authors provide a list of excluded studies and justify the exclusions?*

*Question 8 (Q8) - Did the review authors describe the included studies in adequate detail?*

*Question 9 (Q9) - Did the review authors use a satisfactory technique for assessing the risk of bias (RoB) in individual studies that were included in the review?*

*Question 10 (Q10) - Did the review authors report on the sources of funding for the studies included in the review?*

*Question 11 (Q11) - If meta-analysis was performed did the review authors use appropriate methods for statistical combination of results?*

*Question 12 (Q12) - If meta-analysis was performed, did the review authors assess the potential impact of RoB in individual studies on the results of the meta-analysis or other evidence synthesis?*

*Question 13 (Q13) - Did the review authors account for RoB in individual studies when interpreting/discussing the results of the review?*

*Question 14 (Q14) - Did the review authors provide a satisfactory explanation for, and discussion of, any heterogeneity observed in the results of the review?*

*Question 15 (Q15) - If they performed quantitative synthesis did the review authors carry out an adequate investigation of publication bias (small study bias) and discuss its likely impact on the results of the review?*

*Question 16 (Q16) - Did the review authors report any potential sources of conflict of interest, including any funding they received for conducting the review?*

References

Ahmad, M. S., Shaik, R. A., Ahmad, R. K., Yusuf, M., Khan, M., Almutairi, A. B., et al. (2021). « LONG COVID »: an insight. *Eur. Rev. Med. Pharmacol. Sci.* 25, 5561‑5577. doi: 10.26355/eurrev_202109_26669.

Bertuccelli, M., Ciringione, L., Rubega, M., Bisiacchi, P., Masiero, S., et Del Felice, A. (s. d.). Cognitive impairment in people with previous COVID-19 infection: A scoping review.

Davis, H. E., Assaf, G. S., McCorkell, L., Wei, H., Low, R. J., Re’em, Y., et al. (2021). Characterizing long COVID in an international cohort: 7 months of symptoms and their impact. *EClinicalMedicine* 38, 101019. doi: 10.1016/j.eclinm.2021.101019.

De Luca, P., Di Stadio, A., Colacurcio, V., Marra, P., Scarpa, A., Ricciardiello, F., et al. (2022). Long COVID, audiovestibular symptoms and persistent chemosensory dysfunction: a systematic review of the current evidence. *Acta Otorhinolaryngol. Ital.* 42, S87‑S93. doi: 10.14639/0392-100X-suppl.1-42-2022-10.

Deer, R. R., Rock, M. A., Vasilevsky, N., Carmody, L., Rando, H., Anzalone, A. J., et al. (2021). Characterizing Long COVID: Deep Phenotype of a Complex Condition. *eBioMedicine* 74, 103722. doi: 10.1016/j.ebiom.2021.103722.

Dirican, E., et Bal, T. (2022). COVID-19 disease severity to predict persistent symptoms: a systematic review and meta-analysis. *Prim. Health Care Res. Dev.* 23, e69. doi: 10.1017/S1463423622000585.

Drukker, M., Weltens, I., Hooijdonk, C. F. M. Van, et Vandenberk, E. (2021). Development of a Methodological Quality Criteria List for Observational Studies : The Observational Study Quality Evaluation. 6, 1‑10. doi: 10.3389/frma.2021.675071.

Fernández-de-Las-Peñas, C., Palacios-Ceña, D., Gómez-Mayordomo, V., Florencio, L. L., Cuadrado, M. L., Plaza-Manzano, G., et al. (2021). Prevalence of post-COVID-19 symptoms in hospitalized and non-hospitalized COVID-19 survivors: A systematic review and meta-analysis. *Eur. J. Intern. Med.* 92, 55‑70. doi: 10.1016/j.ejim.2021.06.009.

Girón Pérez, D. A., Fonseca-Agüero, A., Toledo-Ibarra, G. A., Gomez-Valdivia, J. de J., Díaz-Resendiz, K. J. G., Benitez-Trinidad, A. B., et al. (2022). Post-COVID-19 Syndrome in Outpatients and Its Association with Viral Load. *Int. J. Environ. Res. Public Health* 19. doi: 10.3390/ijerph192215145.

Jamoulle, M., Kazeneza-Mugisha, G., et Zayane, A. (2022). Follow-Up of a Cohort of Patients with Post-Acute COVID-19 Syndrome in a Belgian Family Practice. *Viruses* 14, 2000. doi: 10.3390/v14092000.

Malik, P., Patel, K., Pinto, C., Jaiswal, R., Tirupathi, R., Pillai, S., et al. (2022). Post-acute COVID-19 syndrome (PCS) and health-related quality of life (HRQoL)-A systematic review and meta-analysis. *J. Med. Virol.* 94, 253‑262. doi: 10.1002/jmv.27309.

Mendes Paranhos, A. C., Nazareth Dias, Á. R., Machado da Silva, L. C., Vieira Hennemann Koury, G., de Jesus Sousa, E., Cerasi, A. J., et al. (2022). Sociodemographic Characteristics and Comorbidities of Patients With Long COVID and Persistent Olfactory Dysfunction. *JAMA Netw. open* 5, e2230637. doi: 10.1001/jamanetworkopen.2022.30637.

Michelutti, M., Furlanis, G., Buoite Stella, A., Bellavita, G., Frezza, N., Torresin, G., et al. (2022). Sex-dependent characteristics of Neuro-Long-COVID: Data from a dedicated neurology ambulatory service. *J. Neurol. Sci.* 441, 120355. doi: 10.1016/j.jns.2022.120355.

Parker, A. M., Brigham, E., Connolly, B., McPeake, J., Agranovich, A. V, Kenes, M. T., et al. (2021). Addressing the post-acute sequelae of SARS-CoV-2 infection: a multidisciplinary model of care. *Lancet. Respir. Med.* 9, 1328‑1341. doi: 10.1016/S2213-2600(21)00385-4.

Pinzon, R. T., Wijaya, V. O., Jody, A. Al, Nunsio, P. N., et Buana, R. B. (2022). Persistent neurological manifestations in long COVID-19 syndrome: A systematic review and meta-analysis. *J. Infect. Public Health* 15, 856‑869. doi: 10.1016/j.jiph.2022.06.013.

Premraj, L., Kannapadi, N. V., Briggs, J., Seal, S. M., Battaglini, D., Fanning, J., et al. (2022). Mid and long-term neurological and neuropsychiatric manifestations of post-COVID-19 syndrome: A meta-analysis. *J. Neurol. Sci.* 434, 120162. doi: 10.1016/j.jns.2022.120162.

Ser, M. H., Çalıkuşu, F. Z., Tanrıverdi, U., Abbaszade, H., Hakyemez, S., Balkan, İ. İ., et al. (2022). Autonomic and neuropathic complaints of long-COVID objectified: an investigation from electrophysiological perspective. *Neurol. Sci.* 43, 6167‑6177. doi: 10.1007/s10072-022-06350-y.

Shea, B. J., Reeves, B. C., Wells, G., Thuku, M., Hamel, C., Moran, J., et al. (2017). AMSTAR 2 : a critical appraisal tool for systematic reviews that include randomised or non-randomised studies of healthcare interventions , or both. 1‑9. doi: 10.1136/bmj.j4008.

Silva Andrade, B., Siqueira, S., de Assis Soares, W. R., de Souza Rangel, F., Santos, N. O., dos Santos Freitas, A., et al. (2021). Long-COVID and Post-COVID Health Complications: An Up-to-Date Review on Clinical Conditions and Their Possible Molecular Mechanisms. *Viruses* 13, 700. doi: 10.3390/v13040700.

Tan, B. K. J., Han, R., Zhao, J. J., Tan, N. K. W., Quah, E. S. H., Tan, C. J., et al. (2022). Prognosis and persistence of smell and taste dysfunction in patients with covid-19: meta-analysis with parametric cure modelling of recovery curves. *BMJ*, e069503. doi: 10.1136/bmj-2021-069503.

Vandersteen, C., Payne, M., Dumas, L.-E., Metelkina-Fernandez, V., Plonka, A., Chirio, D., et al. (2021). Persistent olfactory complaints after COVID-19: a new interpretation of the psychophysical olfactory scores. *Rhinol. Online* 4, 66‑72. doi: 10.4193/RHINOL/21.010.

Xydakis, M. S., Albers, M. W., Holbrook, E. H., Lyon, D. M., Shih, R. Y., Frasnelli, J. A., et al. (2021). Post-viral effects of COVID-19 in the olfactory system and their implications. *Lancet Neurol.* 20, 753‑761. doi: 10.1016/S1474-4422(21)00182-4.
